# Renal dysfunction, restrictive left ventricular filling pattern and mortality risk in patients admitted with heart failure: a 7-year follow-up study

**DOI:** 10.1186/1471-2369-14-267

**Published:** 2013-12-03

**Authors:** Morten Schou, Jesper Kjaergaard, Christian Torp-Pedersen, Christian Hassager, Finn Gustafsson, Dilek Akkan, Jacob E Moller, Lars Kober

**Affiliations:** 1 Department of Cardiology, The Heart Centre and University of Copenhagen, Rigshospitalet, DK-2100 Copenhagen, Denmark; 2 Department of Cardiology and Endocrinology, Hillerod University Hospital, DK-3400 Hillerod, Denmark; 3 Department of Cardiology, Gentofte University Hospital, DK-2600 Hellerup, Denmark

**Keywords:** Estimated glomerular filtration rate, Restrictive filling pattern, Heart failure, Mortality risk

## Abstract

**Background:**

Renal dysfunction is associated with a variety of cardiac alterations including left ventricular (LV) hypertrophy, LV dilation, and reduction in systolic and diastolic function. It is common and associated with an increased mortality risk in heart failure (HF) patients. This study was designed to evaluate whether severe diastolic dysfunction contribute to the increased mortality risk observed in HF patients with renal dysfunction.

**Methods:**

Using Cox Proportional Hazard Models on data (N = 669) from the EchoCardiography and Heart Outcome Study (ECHOS) study we evaluated whether estimated glomerular filtration rate (eGFR) was associated with mortality risk before and after adjustment for severe diastolic dysfunction. Severe diastolic dysfunction was defined by a restrictive left ventricular filling pattern (RF) (=deceleration time < 140 ms) by Doppler echocardiography.

**Results:**

Median eGFR was 58 ml/min/1.73 m^2^, left ventricular ejection fraction was 33% and RF was observed in 48%. During the 7 year follow up period 432 patients died. Multivariable adjusted eGFR was associated with similar mortality risk before (Hazard Ratio(HR)_eGFR 10 ml increase_: 0.94 (95% CI: 0.89-0.99, P = 0.024) and after (HR_eGFR 10 ml increase_: 0.93 (0.89-0.99), P = 0.012) adjustment for RF (HR: 1.57 (1.28-1.93), P < 0.001).

**Conclusions:**

In patients admitted with HF RF does not contribute to the increased mortality risk observed in patients with a decreased eGFR. Factors other than severe diastolic dysfunction may explain the association between renal function and mortality risk in HF patients.

## Background

An association between renal dysfunction and mortality risk has been documented several times in heart failure (HF) patients [1-6]. The cause of excess mortality in these patients is, however, poorly understood as is the interplay between renal dysfunction and other prognostic factors in HF. Several explanations – e.g. increased atherosclerotic burden, impaired central haemodynamics and increased neurohormonal activity - have been proposed for the increased mortality risk in HF patients with a poor renal function [7-17]. However, an observational follow up study, where all proposed prognostic factors in HF have been included has not yet been conducted in a large HF cohort and consequently it is still unclear if the effect of renal function and mortality risk can be explained by e.g. impaired diastolic function or other important confounders [18].

In patients with chronic kidney disease diastolic dysfunction may be present in up to 70% of the patients [19] and a link between renal dysfunction and left ventricular hypertrophy resulting in diastolic dysfunction may also be present in patients with HF, where renal dysfunction is often present. In theory, renal dysfunction could contribute to the heart failure syndrome, negatively affecting prognosis, by adding the burden of impaired filling to patients suffering from HF. However, studies with long-term follow-up and information on systolic, diastolic and renal function are lacking in HF patients.

To address these issues we used data from the EchoCardiography and Heart Outcome Study (ECHOS) study to evaluate whether the increased mortality risk observed in HF patients with renal dysfunction potentially could be explained by severe diastolic dysfunction.

## Methods

### Study population and patient selection

The ECHOS study was a trial of the central symphatolytic agent CHF1035 versus placebo in patients with HF. The trial design and results have been described in detail previously [20,21]. Briefly, from 2001 to 2002, 3954 consecutive patients admitted with congestive HF were screened in 43 hospitals in Scandinavia, primarily Denmark. Congestive HF was defined as current NYHA functional class II–IV, requiring intravenous treatment with diuretic and at least one episode of dyspnoea or fatigue at rest or during slight exertion within the last month corresponding to at least NYHA class III or IV). Demographic variables and comorbid conditions were registered (Table 1). A Wall Motion Index (WMI) ≤ 1.2 (≈ Left ventricular ejection fraction (LVEF) ≤ 35%) was found in 1831 patients and 1000 patients were eventually randomized in the drug trial. Patients were randomized to either placebo (N = 499) or a selective agonist of the pre-synaptic DA2- and α2-receptors (N = 501) and were followed for at least 12 months. The study drug had no effect on overall mortality or morbidity [20]. The study was carried out in accordance with the Declaration of Helsinki II and the study was approved by The Ethical Committee of Copenhagen, Denmark. The current study is based on data obtained from screening of patients admitted with HF to the departments participating in the ECHOS study. Hence, this study included both patients with systolic and diastolic HF.

**Table 1:** Patients characteristics according to groups of eGFR (N = 669)

**Demographic data**	**eGFR: < 45.0 ml/min 1.73 m**^**2**^ **N = 191**	**eGFR: 45.0-59.9 ml/min 1.73 m**^**2**^ **N = 162**	**eGFR: 60.0 – 74.9 ml/min 1.73 m**^**2**^ **N = 176**	**eGFR: ≥75.0 ml/min1.73 m**^**2**^ **N = 140**	**P value**
**Clinical variables:**					
Age, years	78 (63–91)	75 (56–89)	72 (55–90)	68 (46–84)	<0.001
Female sex,%	45	43	30	34	0.006
BMI, kg/m^2^	25 (19–26, 26–36)	26 (17–37)	26 (18–34)	27 (20–41)	0.058
Smoking,%	20	23	31	43	<0.001
History of HF,%	76	76	63	54	<0.001
NYHA Class,%:					0.052
I	7	9	15	15	
II	65	65	62	65	
III	25	25	19	20	
IV	3	1	4	0	
**Comorbidity:**					
Diabetes,%	22	15	13	15	0.049
COPD,%	20	19	23	26	0.111
IHD,%	54	58	45	39	0.002
Previous MI,%	32	35	30	25	0.139
Hypertension,%	32	32	25	31	0.484
Chronic AF,%	26	21	28	19	0.313
Paroxysmal AF,%	19	22	21	21	0.801
**Echocardiographic variables:**					
WMI, index:	1.2 (0.5–2.0)	1.1 (0.5–2.0)	1.3 (0.5–2.0)	1.4 (0.6–2.0)	0.093
LVEDD, mm	57 (42–75)	59 (41–77)	58 (42–70)	57 (42–78)	0.878
A, m/s	0.63 (0.30–1.15)	0.76 (0.30–1.33)	0.62 (0.32–1.03)	0.62 (0.33–0.96)	0.055
E/A-ratio	1.34 (0.56–3.37)	1.11 (0.57–3.11)	1.12 (0.58–3.03)	1.40 (0.60–3.12)	0.4639
**Medication (discharge):**					
ACE-I,%	51	70	63	45	0.309
ARB,%	10	9	5	9	0.266
Beta blockers,%	49	57	45	42	0.097
Diuretics,%	95	93	89	89	0.017
Digoxin,%	37	39	47	36	0.634
Statin,%	17	20	16	23	0.470
Nitrates,%	28	23	12	17	0.001

eGFR = estimated glomerular filtration rate; BMI = Body Mass Index; History of HF = history of heart failure; NYHA class = New York Heart Association; COPD = chronic obstructive pulmonary disease; IHD = ischemic heart disease; Previous MI = previous myocardial infarction; Chronic AF = chronic atrial fibrillation; Paroxysmal AF = paroxysmal atrial fibrillation; WMI = wall motion index; LVEDD = left ventricular enddiastolic diameter; A = late atrial mitral Doppler peak flow velocity; E/A-ratio = the ratio of early (E) to late atrial (A) mitral Doppler peak flow velocity; ACE-I = angiotensin converting enzyme inhibitor; ARB = angiotensin II receptor blocker.

For the present analyses, patients were selected as follows: Of the initial 3954 patients screened for the ECHOS study, only Danish patients were available for the extended follow up (N = 3078). Of these patients 2881 patients had measurement of LVEF. Both LVEF and serum(s)-creatinine were available in 2012 patients. S-creatinine together with left ventricular filling pattern obtained by the flow velocity across the mitral valve in the apical 4-chamber view were available in 669 patients (Table 1). Included (N = 669) and excluded (N = 2212) patients were subsequently compared concerning important demographic variables (age, sex, NYHA class, WMI, eGFR, frequency of RF (available in 967 patients), and mortality risk (see Results)).

### Echocardiography

Echocardiography was performed within the first 7 days after admission. Patient history and a physical examination were obtained and all patients underwent 2-dimensional baseline transthoracic echocardiography in order to determine LVEF. In addition to standard 2-dimensional echocardiography the centers were also encouraged to perform Doppler ultrasound and M-mode examinations on a voluntary basis. Doppler flow recordings of sufficient quality were available for 972 patients. Echocardiograms were digitized and subsequently analyzed by two experienced investigators (D.A. and J.K.) who were blinded to all clinical data. Analyses of wall motion index (WMI) were evaluated separately in a core lab. LVEF was assessed semi-quantitatively from WMI using a 16-segment model. Using a reverse scoring system, each of the 16 left ventricular segments were assigned a descending score from 3 to −1, according to myocardial function (hyperkinesis = 3, normokinesis = 2, hypokinesis = 1, akinesis = 0, paradoxical motion = −1). WMI was calculated by dividing the sum of the scores by the number of segments analyzed. Left ventricular filling pattern was obtained by measuring the diastolic flow velocities across the mitral valve in the apical 4- chamber view. Effort was made to detect maximal flow velocities during diastole with the sample volume placed at the tip of the mitral leaflets. Deceleration time of early filling (DT), peak flow velocity in early diastole (E velocity) and peak flow velocity at atrial contraction (A velocity) were measured as the averages of five consecutive cardiac cycles. DT was measured similarly in patients with atrial fibrillation/-flutter, but was based on averages of 5 to 10 consecutive cardiac cycles. Cardiac cycles with fusion of early and late velocities or with nonlinear deceleration slope were excluded. RF was considered present when deceleration time was ≤140 ms [21].

### Estimated glomerular filtration rate

Estimated glomerular filtration rate (eGFR) was calculated by the four-component Modification of Diet in Renal Disease (MDRD) equation incorporating age, race, sex and s-creatinine concentration [16]: eGFR = 186 * (s-creatinine [in milligrams per decilitre])^-1.154^ * (age [in years])^-0.203^. For women the product of the equation has to be multiplied by a correction factor of 0.742. The equation has been evaluated in heart failure patients [22].

### End points

The primary end-point was death from all causes obtained from the Danish Central Personal Registry, where all deaths in Denmark are registered within 2 weeks. Thus, in (May 2008) the central registry provided 7 years (median) of follow-up (range: 5.8 to 7.4 years) for patients included in the current analyses. One patient emigrated and was censored at time of emigration (after 472 days), no other patients were lost to follow-up.

### Statistics

Patients, were grouped by levels of eGFR: Group I: eGFR < 45.0 ml/min/1.73 m^2^, Group II: eGFR 45.0-59.9 ml/min/1.73 m^2^, Group III: eGFR 60.0-74.9 ml/min/1.73 m^2^ and Group IV: eGFR ≥ 75.0 ml/min/1.73 m^2^. Proportions are presented as percent and continuous variables are presented as medians with 95% confidence intervals. Baseline characteristics were compared between groups using chi-square test for discrete variables and, Cochran-Armitage Trend Test, Kruskal Wallis tests (non-parametric) or one-way ANOVA (parametric) for continuous variables, as appropriate. The associations between eGFR and mortality were examined using Cox proportional hazards model. Survival curves were generated by means of Kaplan-Meier estimates, and differences in survival were compared using log-rank test. Cox proportional multivariate hazard models were fitted with the use of available clinical covariates (eGFR, age, sex, body mass index, smoking, RF, WMI, NYHA class, diabetes, history of hypertension, history of chronic obstructive pulmonary disease, history of ischemic heart disease, previous MI, and atrial fibrillation). The association between eGFR and mortality risk was evaluated by a univariate analysis (model 1), after adjustment for all covariates except RF (model 2) and after adjustment for all covariates including RF (model 3). The association between RF and mortality risk was evaluated in a similar way (model 4–6). The assumptions underlying the Cox proportional-hazards model (proportional hazards, lack of interaction, and linearity of continuous variables) were tested and found to be valid. A P-value < 0.05 was considered significant (two-sided). Analyses were performed using Statistical Analysis Software (SAS 9.1, Cary, NC, USA).

## Results

### Patients characteristics

**Figure 1:**
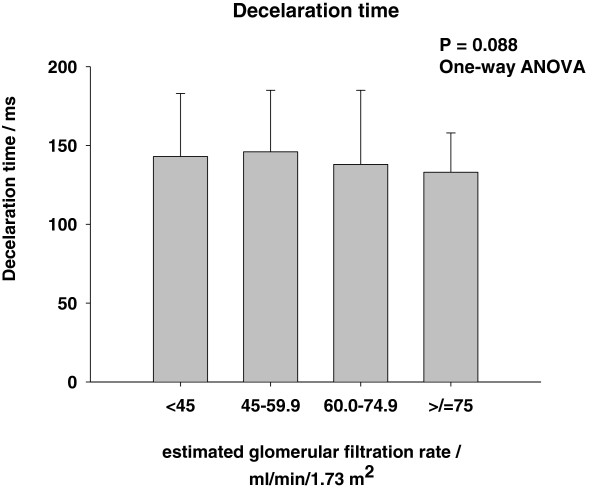
Deceleration time.

**Figure 2:**
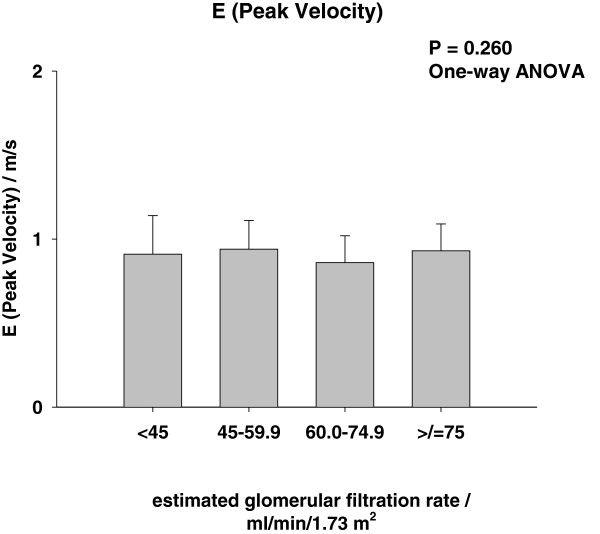
E (peak velocity) and frequency of restrictive filling pattern.

**Figure 3:**
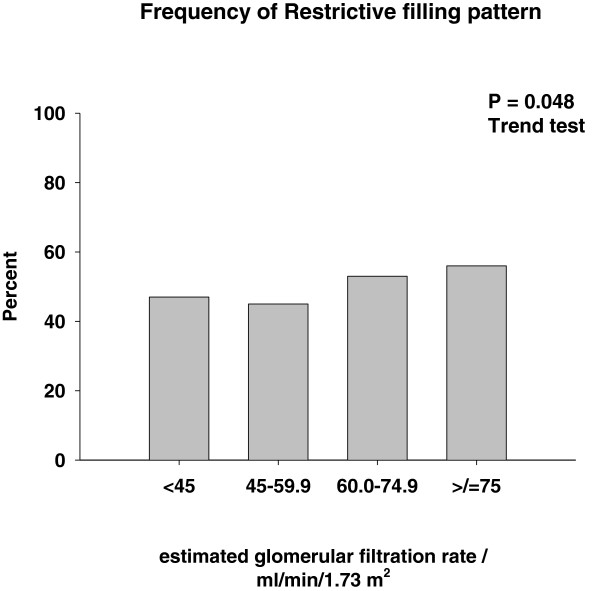
**According to groups of estimated glomerular filtration rate (Group I: eGFR < 45 ml/min/1.73 m**^**2**^**, Group II: eGFR 45.0-59.9 ml/min/1.73 m**^**2**^**, Group III: eGFR 60–74.9 ml/min/1.73 m**^**2 **^**and Group IV: eGFR >/= 75.0 ml/min/1.73 m**^**2**^).*Median and interquartil range.

Patient characteristics according to groups of eGFR are presented in Table 1 and Figures 1, 2, 3. Patients with a low eGFR (group IV) were older (P < 0.001), more frequently female (P < 0.001), and less frequently smokers (P < 0.001). More frequently patients with low eGFR suffered from diabetes (P = 0.049), had a history of HF (P < 0.001) and ischemic heart disease (P = 0.002). Finally, patients with a low eGFR were treated with nitrates (P = 0.001) and diuretics (P = 0.017) more frequently. None of the Doppler parameters were affected by eGFR (Figures 1, 2, 3), except the prevalence of RF (P = 0.049), which increased with increasing eGFR (Figure 3).

### Included (N = 669) versus excluded (N =2212) patients

The included and excluded patients did not differ with regard to age (P = 0.36), sex (P = 0.54), eGFR (P = 0.23) and NYHA class (P = 0.11), but differed to a minimal extent with regard to the frequency of left ventricular systolic dysfunction (41% of the included patients had a LVEF < 0.50 compared to 35% of the excluded patients (P = 0.003)), which may be explained by the fact that s-creatinine was reported more frequently in the randomized patients.

### Survival data

**Figure 4:**
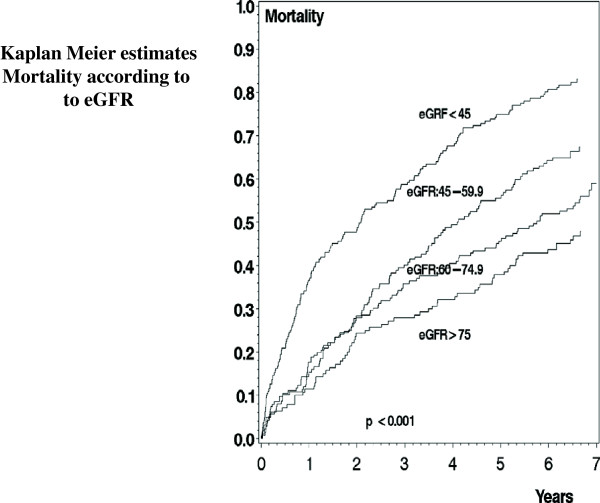
**Kaplan Meier estimates according to groups of estimated glomerular filtration rate (Group I: eGFR < 45 ml/min/1.73 m**^**2**^**, Group II: eGFR 45.0-59.9 ml/min/1.73 m**^**2**^**, Group III: eGFR 60–74.9 ml/min/1.73 m**^**2 **^**and Group IV: eGFR >/= 75.0 ml/min/1.73 m**^**2**^**).**

Kaplan-Meier plots showing mortality rates according to groups of eGFR are presented in Figure 4 (LogRank P < 0.001). In the follow-up period 432 patients died.

Univariate and multivariable Cox proportional hazard models are presented in Table 2. Adjustment for RF (model 4 versus model 5) did not affect the parameter estimates for eGFR suggesting that the association between eGFR and mortality risk is not confounded by RF. Nor was the opposite the case (model 2 versus model 5) suggesting that nor is the association between RF and mortality risk confounded by eGFR. We did not observe any statistical interaction between eGFR, RF and mortality risk (P > 0.05) nor was any interaction between mortality risk, eGFR < 30 ml/min/1.73 m^2^ and RF (P > 0.05) observed. Furthermore, we did not observe any interaction between mortality risk, RF and ischemic versus non-ischemic cardiomyopathy (P > 0.05), between mortality risk, RF and history of hypertension (P > 0.05) and between mortality risk, RF and LVEF > 40% (P > 0.05). The prognostic significance of RF did, therefore, not interact with any of these important subgroups for which reason the patients were not divided further into subgroups.

**Table 2:** Multivariate cox proportional hazard models

**Variable**	**Hazard ratio (95% confidence interval)**	**P value**
**Model 1:** eGFR (Univariate)	−2LogL: 5172	
eGFR_effect of a 10 ml increase_	0.83 (0.79-0.87)	<0.001
**Model 2:** eGFR + traditional confounders	−2LogL: 4614	
eGFR_effect of a 10 ml increase_	0.94 (0.89-0.99)	0.024
**Model 3:** RF (Univariate)	−2LogL: 5223	
RF	1.38 (1.14-1.66)	<0.001
**Model 4:** RF + traditional confounders	−2LogL: 4601	
RF	1.54 (1.26-1.89)	<0.001
**Model 5:** eGFR + RF + traditional confounders	−2LogL: 4595	
eGFR_effect of a 10 ml increase_	0.93 (0.89-0.99)	0.012
RF	1.57 (1.28-1.93)	<0.001

Significant traditional confounders: age, sex, wall motion index, ischemic heart disease, diabetes, chronic obstructive pulmonary disease and NYHA class. Covariates were removed by backward elimination if P > 0.10.

## Discussion

Our data suggest that eGFR is associated with mortality risk independently of RF in HF patients. We did not observe any coupling between renal function and severe diastolic dysfunction and likely other factors than impaired left ventricular filling explain the association between renal function and mortality risk in HF patients.

Previously, it has been observed that eGFR adds prognostic information to RF in outpatients with chronic HF and systolic dysfunction [23]. However, our data differ from the data by Bruch et al. [23] since we investigated patients admitted for acute decompensated heart failure with preserved and reduced LVEF and selected death as endpoint, in contrast to Bruch et al. [23] that selected a composite endpoint of death, admission and heart transplantation.

It has been repeatedly documented that eGFR is associated with increased long term mortality risk in patients with HF [1-6], but adjustment for diastolic function has not been performed in previous studies. Data from the DIG-trial has shown that renal dysfunction carried incremental prognostic information in HF patients with preserved LVEF, but diastolic function was not recorded [6]. We observed that adjustment for RF did not affect the parameter estimates for eGFR (Table 2). Consequently, RF cannot explain the excess mortality associated with renal dysfunction. Early mitral inflow peak velocity (E), E/A-ratio and DT did not correlate to eGFR (Figures 1, 2 and Table 1), but the frequency of RF increased slightly with increasing eGFR (Figure 3). Accordingly, the severity of diastolic dysfunction did not increase with decreasing eGFR. This observation may reflect that RF alone does not reflect severity of diastolic dysfunction and intracardiac pressures accurately in this population [24] or that a diuresed HF population with a high prevalence of renal dysfunction is different from a chronic kidney disease population with cardiac dysfunction [19]. It could, of course also be a consequence of a type II error or selection bias. Lending indirect support to the recorded left ventricular filling patterns is the fact that symptom severity as assessed by NYHA class also was unaffected by renal function (Table 1) [25]. Finally, we did not observe any link between eGFR and RF in patients admitted with acute decompensated HF (Cardio-renal syndrome Type I). However, this observation does not exclude and association in e.g. acute and chronic reno-cardiac syndromes as previously observed [19].

Despite the close association with morality risk no randomized clinical trials have indicated that an intervention raised against renal dysfunction in HF can improve outcome. In acute decompensated heart failure ultrafiltration has been suggested to prevent deterioration of eGFR [26], however, it remains to be determined whether this improve long-term clinical outcome. Recently, despite encouraging early results, an adenosine receptor antagonist (Rolofylline) failed to improve renal function in HF patients [27] and the role for nesiritide is also unclear [28]. However, one non-randomized trial of a systematic diagnostic and therapeutic approach directed against renal dysfunction in HF patients has indicated that it is possible to obtain a significant improvement in kidney function [29], and in patients, where cardiac resynchronization therapy is indicated, this intervention may improve renal function [30]. Clearly the issue is complex. In some instances, such as during uptitration of ACE-inhibitors or angiotensin II receptor blockers, transient increase in s-creatinine is expected and accepted due to the well known long term benefit in terms of reduced morbidity and mortality. Thus, data suggest deterioration of renal function in HF are associated with a poor outcome, but optimal management depends on the clinical setting e.g. episodes of decompensation and hyperkalemia versus up-titration of an angiotensin converting inhibitor [31].

Based on the data from the present study as well as results of previous studies, eGFR may be considered independently associated with mortality risk in HF patients. The underlying mechanism is not clear. In particular, it is uncertain if low eGFR is associated with mortality mainly due to a renal factor (complicating intrinsic renal disease) or mainly due to the fact that low eGFR reflects low renal perfusion pressure because of poor cardiac output (cardiac factor in turn leading to poor prognosis). Although the present study suggest that the adverse prognosis associated with low GFR is largely unrelated to low LVEF or advanced diastolic dysfunction, the data cannot determine whether the excess mortality is related to an intrinsic renal mechanism or merely reflect a more extensive vascular disease and thus increased risk in these patients. It may be speculated that activation of the renin-angiotensin-aldosterone-system (RAAS) causes progression of renal dysfunction in HF similar to the development of diabetic nephropathy, which is supported by the CATS study (post myocardial infarction) [32]. However, aggressive blockade of the Renin-angiotensin II-aldosterone system (RAAS) in HF may lead to worsening renal function as seen in the CHARM-added [33], VALHeft [34] and VALLIANT [35] studies. A better understanding of the effect of RAAS inhibition on other renal endpoints like micro- and macroalbuminuria in HF is clearly needed [16,36,37].

Attention should be paid to some methodological limitations of the present study. Our analyses are retrospective, but it seems unlikely that the observed associations between eGFR or RF and mortality risk reflect type I errors. Left ventricular filling pattern and s-creatinine data was only available in 669 out of 2881 patients and it may therefore be argued that our results reflect selection bias. However, the included patients did not differ from the excluded patients with respect to important clinical variables (internal validity acceptable) and selection bias would only appear if the associations between eGFR and RF or the associations between eGFR or RF and mortality risk were different in the excluded patients, which seems unlikely. Furthermore, the prognostic significance of RF and eGFR is in accordance with other studies (external validity acceptable) [1,38,39]. We used eGFR as estimate for GFR, and we did not analyze s-creatinine in a core lab, and this could lead to analytical variation. Furthermore, we defined severe diastolic dysfunction solely by RF and do not have data on left atria volume, left ventricular hypertrophy or tissue Doppler variables and it may therefore be argued that our results reflect inaccurate measurement of GFR and misclassification of severe diastolic dysfunction. However, recently it has been questioned whether a gold standard for diastolic function exists at all [40] and RF may be a reasonable estimate due to its extensive documented association with mortality risk in patients with HF and myocardial infarction [35,36]. Though, misclassification of severe diastolic dysfunction may have occurred due to lack of data on left atria volume, left ventricular hypertrophy and tissue Doppler. Patients with HF and preserved ejection (35% of the present patients) constitute an inhomogeneous group and it may be argued that our results to some degree could reflect misclassification of HF [41]. The generalizability of our results should, therefore, be noticed. The present cohort neither reflects a cohort of acute decompensated HF patients with a reduced LVEF which are e.g “cold and wet” nor a cohort of patients with concentric left ventricular hypertrophy, normal LVEF and HF symptoms, rather it is a cohort of patients with dyspnoea requiring diuretics (“warm and wet”). The strengths of our study are the relatively large sample size with data on both eGFR and RF, the inclusion of patients with s-creatinine data > 200 umol/L (data from a screening database and not from a trial database), the long term follow up period without any lost to follow up and the large number of events.

## Conclusions

In HF patients restrictive filling of the left ventricle does not explain the increased mortality risk observed with decreasing eGFR. Factors other than impaired left ventricular filling explain the association between renal dysfunction and mortality risk. Whether the effect of eGFR and mortality risk in HF *per se* is causal deserves further study as does the association between eGFR and diastolic function in HF.

## Competing interests

The authors declare that they have no competing interests.

## Authors’ contributions

MS: hypothesis, data analysis and manuscript preparation; JK: data collection, data analysis and manuscript preparation; CTP: data collection, data analysis and manuscript preparation; CH: data collection, data analysis and manuscript preparation; FG: hypothesis, data analysis and manuscript preparation; DA: data collection, data analysis and manuscript preparation; JEM: data collection, data analysis and manuscript preparation; LK: hypothesis, data collection, data analysis and manuscript preparation. All authors read and approved the final manuscript.

## Pre-publication history

The pre-publication history for this paper can be accessed here:

http://www.biomedcentral.com/1471-2369/14/267/prepub
